# Magnetic resonance imaging for paediatric retroperitoneal masses: Diagnostic accuracy of the claw sign

**DOI:** 10.4102/sajr.v25i1.2012

**Published:** 2021-02-26

**Authors:** Lisa Combrink, Kenneth B. Beviss-Challinor

**Affiliations:** 1 Department of Radiology, Faculty of Medicine and Health Sciences, Stellenbosch University, Cape Town, South Africa; 2 Department of Radiodiagnosis, Faculty of Medicine and Health Sciences, Stellenbosch University, Cape Town, South Africa

**Keywords:** claw sign, diagnostic accuracy, radiology, radiodiagnosis, paediatrics

## Abstract

**Background:**

The claw sign is advocated as a discriminant of renal versus non-renal origin of tumours. The accuracy of the claw sign on magnetic resonance imaging (MRI) is unknown and is potentially hindered by the inferior spatial resolution and the larger tumour sizes at presentation in developing countries.

**Objectives:**

To define and evaluate the claw sign in differentiating renal from non-renal retroperitoneal masses in children undergoing MRI.

**Methods:**

A definition of the claw sign was proposed. Magnetic resonance imaging studies, clinical and laboratory records of 53 children were reviewed to test the diagnostic accuracy, inter- and intra-observer reliability. Three tumour–mass interface characteristics, inherent to the claw sign, were tested: (1) a smooth tapering kidney edge blending continuously with the tumour, (2) absence of infolding of the kidney and (3) an obtuse superficial angle.

**Results:**

The sensitivity, specificity, negative predictive value and positive predictive values of the claw sign were 97%, 74%, 83% and 94%. The Cohen’s kappa values for intra-rater reliability were 0.72 (95% confidence interval 0.54–0.86) for the first reader and 0.83 (0.66–1.00) for the second reader. The Cohen’s kappa values for inter-rater reliability were 0.67 (0.50–0.85) and 0.65 (0.44–0.86) for the second reading respectively (*p* < 0.0001).

**Conclusion:**

The three tumour–mass interface characteristics investigated are all important characteristics of the claw sign. Intra- and inter-rater reliability is moderate to strong for all characteristics and overall impression of the claw sign. The claw sign is therefore sensitive in the accurate placement of an intra-renal mass but lacks specificity.

## Introduction

Childhood cancer is rare, with 600–700 new cases reported per year for the past 25 years and entered in the South African Children’s Tumour Registry.^1^ The majority of abdominal malignancies in children are of retroperitoneal origin.^2^ Nephroblastoma, a malignancy of renal origin, and neuroblastoma, usually an extra-renal retroperitoneal malignancy, together accounted for 1217 (18%) of all South African childhood cancers in the decade ending 2007.^1^ Both malignancies are potentially curable, although the frequently advanced stage of presentation in South Africa drives overall survival rates inferior to those reported in developed countries.^1^

Imaging plays a pivotal role in the diagnosis and management pathway of a child presenting with an abdominal mass. Accurate diagnosis is paramount, as different malignancies have different clinical management strategies and prognoses. A crucial step in narrowing the differential diagnosis involves determining whether the tumour is renal or extra-renal in origin.^3^ This differentiation is not always routinely apparent, particularly when faced with large masses. Along with other findings, the absence or presence of a claw sign is widely advocated as a useful discriminator to determine the origin of a mass.^4^

The origins of the claw sign are uncertain. Furthermore, previous descriptions are insufficiently explicit, leading to potential ambiguity in interpretation and possibly contributing to the limited evidence base for its diagnostic accuracy. There is no guidance as to which modalities or imaging planes provide for optimal assessment, or whether the sign should be identified on single or multiple tumour–kidney interfaces to be called positive.

We understand the claw sign to be positive when a rim of renal parenchyma extends around a mass, resembling a lobster claw in appearance. The tumour–mass interface typically has three characteristics: a smooth tapering kidney edge blending continuously with the tumour, absence of rounding or infolding of the kidney and an obtuse superficial angle. A positive claw sign suggests a renal origin of the mass, with its absence suggesting a non-renal origin.

The impact of size on the accuracy of the claw sign is unknown. Intuitively, it seems likely that the claw sign would be more accurate in smaller tumours, where renal anatomy is relatively well-maintained. Unfortunately, retroperitoneal tumours in children are frequently large at presentation, particularly in South Africa.^5^ Larger tumours frequently cause substantial anatomic distortion where critical evaluation of the tumour–kidney interface can prove challenging. This may limit claw sign application and accuracy.^6^

Computed tomography (CT) and magnetic resonance imaging (MRI) demonstrate similar performance in the abdominal staging of paediatric renal malignancies.^7^ In keeping with international trends, MRI is preferred as compared to CT for directing surgical planning and risk stratification in children with retroperitoneal tumours presenting to our hospital.^4^ This is driven by concerns about cancer induction in children, who are more susceptible to stochastic effects of radiation than adults, and the superior soft tissue contrast of MRI. However, the inferior spatial resolution of MRI compared to CT provides a potential diagnostic disadvantage and may impair the accuracy of findings such as the claw sign.

There are no studies validating the diagnostic accuracy of the claw sign on MRI. While the claw sign is interpreted in conjunction with other signs, it is plausible that radiologists may assign an unwarranted significance to it, leading to diagnostic errors. There is also the potential for bias, favouring the reporting of positive claw signs and non-reporting of negative claw signs.

This study aims to describe the application and diagnostic accuracy of the claw sign through an audit of local practice; propose an unambiguous definition of the claw sign and test its accuracy; and establish the intra- and inter-observer agreement and diagnostic accuracy of the claw sign on MRI.

## Research methods and design

### Study design and setting

This retrospective, descriptive study addressed the prevalence and accuracy of the claw sign on abdominal MRI in current Tygerberg Hospital radiology practice and assessed the intra- and inter-rater reliability and diagnostic accuracy of the claw sign. The study was set within the Division of Radiodiagnosis, at a tertiary public hospital in Southern Africa. The study was conducted as an undergraduate research affiliated with the University of Stellenbosch.

All children aged between 0 and 12 years with solid or mixed solid-cystic retroperitoneal masses, undergoing their first MRI examination between 01 January 2013 and 31 December 2018, were included in the study. Cases with no final clinical diagnosis or ambiguous medical, surgical or laboratory records were excluded.

### Data collection and interpretation

The radiological information system database (RIS) of the Tygerberg hospital was accessed to identify all cases available within the established time span. The accompanying referral documents and radiology reports were used to identify the subset of patients with a mass inseparable from the kidney. An electronic search of the radiology report using the keyword ‘claw’ was performed to identify radiology reports containing reference to the claw sign. Clinical and laboratory records were reviewed and accessed using the National Health Laboratory Services (NHLS) ‘Trakcare’ and ‘Enterprise Content Manager’ (ECM) databases. Missing reports were further accessed with aid from the Tygerberg Hospital Division of Anatomical Pathology. Clinical and laboratory records were used to establish the final diagnosis as the gold standard.

Two radiologists, one with 16 years’ experience and the other a registered fifth-year student accredited with having passed the specialty examinations from the Division of Radiodiagnosis at Tygerberg hospital, reviewed the MRI images of the study population and determined the claw sign as present or absent. The radiologists were blinded to referral information, as well as the original radiology report. All MRI scans were performed on a 1.5-Tesla Siemens scanner. Only the axial (repetition time [TR] 5 ms, time to echo [TE] 2.5 ms, slice thickness 5 mm) and coronal (TR 4.5 ms, TE 2.3 ms, slice thickness 4.5 mm) T2-weighted, gradient echo and true fast imaging with steady-state free precession (TRUFI) images were read, as these were considered to best delineate the kidney–tumour interface. A second reading of the images was then taken 1 month later. Inconsistencies with reports were resolved by consensus.

### Data analysis

All data were captured on a Microsoft Excel spreadsheet. Variables recorded included age, tissue diagnosis, tapered kidney edge, infolding of the kidney edge, obtuse external kidney–tumour angle, axial and coronal tumour dimension and presence or absence of the claw sign.

The spreadsheet data were imported using SPSS Statistics for Windows version 25.0 for analysis. Sensitivity, specificity, positive and negative predictive values were calculated by cross tabulating the binary index variables with the gold standard histology. The 95% confidence intervals (CI) were reported around the sample estimates. Axial and coronal tumour dimensions on MRI were examined for their usefulness in predicting gold standard histology using receiver operating characteristic (ROC) curves. The area under the curve and its 95% CIs were reported.

Cohen’s kappa statistics were calculated for both inter- and intra-rater reliability. A *p*-value <0.05 indicated a better reliability than would have occurred by chance alone. The cut-off points used to interpret the kappa values are those documented by McHugh.^8^

### Ethical consideration

The Response to Modifications received on 09 May 2019 was reviewed by members of the Undergraduate Research Ethics Committee (UREC) via Minimal Risk Review procedures on 20 May 2019 and was approved. This study was approved by the Health Research Ethics Committee (HREC), Stellenbosch University, with a waiver of informed consent due to the retrospective descriptive nature of the study (HREC/UREC reference number: U18/10/036). The research study did not entail intervention directly affecting the patient population. There were no anticipated risks to the included patients.

## Results

A total of 53 cases were identified, 22 male and 31 female patients. The age range for the study population was 1 to 108 months (median 24 months, mean 30 months). The mean axial tumour dimension was 9.8 centimetres (cm) with a median of 10.0 cm. The mean coronal tumour dimension was 11.0 cm with a median of 11.4 cm.

**FIGURE 1:**
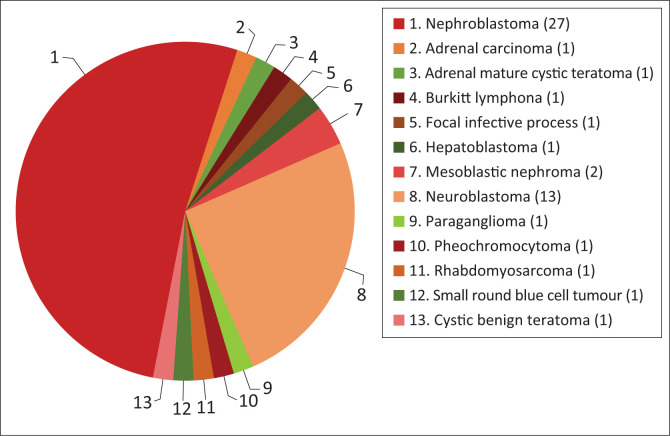
Histological frequency (numbers) of the study population.

Of the 53 cases analysed, 31 (58.5%) were intra-renal and 22 (41.5%) were extra-renal tumours (Figure 1). The intra-renal tumours were nephroblastomas (*n* = 27), mesoblastic nephromas (*n* = 2), a focal infective process (*n* = 1) and cystic benign teratoma (*n* = 1). The extra-renal tumours were neuroblastomas (*n* = 13), rhabdomyosarcomas (*n* = 2), adrenal carcinoma (*n* = 1), adrenal mature cystic teratoma (*n* = 1), Burkitt’s lymphoma (*n* = 1), hepatoblastoma (*n* = 1), paraganglioma (*n* = 1), phaeochromocytoma (*n* = 1) and small round blue cell tumour (*n* = 1) (summarised in Figure 1).

The sensitivity, specificity, positive and negative predictive values of the overall assessment of the claw sign and tumour–mass interface characteristics are presented in Table 1, along with the intra- and inter-rater reliabilities.

**TABLE 1:** Summary table of sensitivity (%), specificity (%), positive predictive value (%), negative predictive value (%), intra-rater and inter-rater reliability of the tumour–mass interface characteristics and the overall assessment of the claw sign.

Tumour–mass interface characteristics	Sensitivity (%)	Specificity (%)	Positive predictive value (%)	Negative predictive value (%)	Intra-rater reliability (first reader)*		Intra-rater reliability (second reader)*		Inter-rater reliability (first reading)*		Inter-rater reliability (second reading)*	
					Cohen’s kappa	95% CI	Cohen’s kappa	95% CI	Cohen’s kappa	95% CI	Cohen’s kappa	95% CI
Tapered kidney edge	97	74	83	94	0.67	0.49–0.85	0.96	0.87–1.00	0.73	0.53–0.88	0.75	0.56–0.94
Absent infolding	97	70	81	94	0.71	0.54–0.89	0.96	0.87–1.00	0.71	0.53–0.86	0.83	0.67–0.99
Obtuse external interface	93	74	82	89	0.74	0.56–0.89	0.96	0.87–1.00	0.69	0.49–0.88	0.71	0.52–0.91
Claw sign	97	74	83	94	0.72	0.54–0.86	0.83	0.66–1.00	0.67	0.50–0.85	0.65	0.44–0.86

CI, confidence interval.

* , *p* < 0.0001.

**FIGURE 2:**
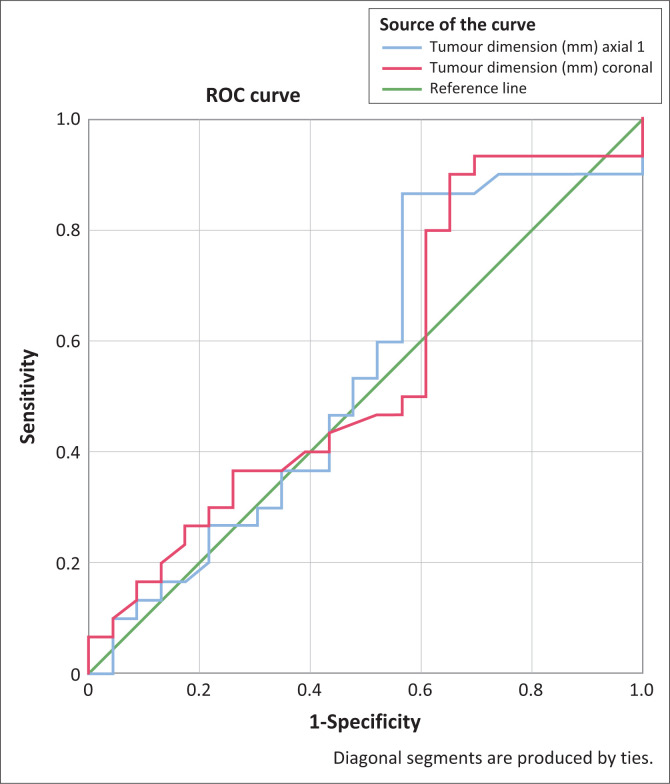
Receiver operating characteristic curve of tumour dimensions on coronal and axial magnetic resonance imaging. ROC, receiver operating characteristic.

The ROC analysis, when using axial tumour dimension on MRI as the quantitative variable, was 0.549 (95% CI: 0.386–0.712). The area under the curve for the coronal tumour dimension on MRI was 0.554 (95% CI: 0.393–0.716; see Figure 2).

## Discussion

To our knowledge, there were no previous studies evaluating the application and diagnostic accuracy of the claw sign on MRI. The claw sign is often advocated as useful in determining whether tumours are of renal or extra-renal origin. Fifty per cent (50.9%) of the study population seen at Tygerberg hospital in the last 5 years proved to have nephroblastomas, with neuroblastomas comprising 25% of the total study population. This highlights the important task faced by radiologists in distinguishing between the intra- or extra-renal origins of a tumour.

In our study population, the claw sign on MRI, proved to be a good predictor of location using histological correlation as the gold standard with high sensitivity (97%) and positive predictive value (94%), but lower specificity (74%) and negative predictive value (83%). The authors anticipated that as tumour size increased, the use of the claw sign would become less accurate and may explain the lower specificity.

The most common presenting symptom in these patients was that of a palpable abdominal mass, implying a moderate to large tumour size at diagnosis.^4^ Large-mass lesions are prone to distort kidney anatomy, making the interpretation of the claw sign difficult. As previously mentioned, there is no guidance as to which modalities or imaging planes provide for optimal assessment of the claw sign, or whether the manifestation should be identified on single or multiple tumour–kidney interfaces to be called positive. The average tumour dimension in our study (9.8 cm) corresponded to the reported value of 9.82 cm in the study by Wu et al.^6^ which suggests that the large tumour size at presentation may not be a problem limited to the South African context.

According to the ROC analysis, the area under the curve of both the axial and coronal tumour dimension was low (0.549 and 0.544, respectively). There is therefore no preferential anatomical plane in which to assess for the claw sign as both quantitative variables perform equally poorly. Neither was a size threshold at which the claw sign optimally performed, established. This may be due to the limitation of the study population, interpreter dependence or unknown reporting bias.

The following three characteristics were evaluated for final diagnosis of a positive claw sign: renal mass displaying a tapered kidney edge, absent infolding of the kidney edge and an obtuse external kidney–tumour interface. The intra-rater reliability for each of these signs was substantial for reader one (Cohen’s kappa values of 0.67, 0.71 and 0.74, respectively) and very strong for reader two (0.96 for all three). The inter-rater reliability was substantial for the first read (0.73, 0.71 and 0.69) and very similar to the second read (0.75, 0.83 and 0.71, Table 1), indicating a very high degree of consensus.

**FIGURE 3:**
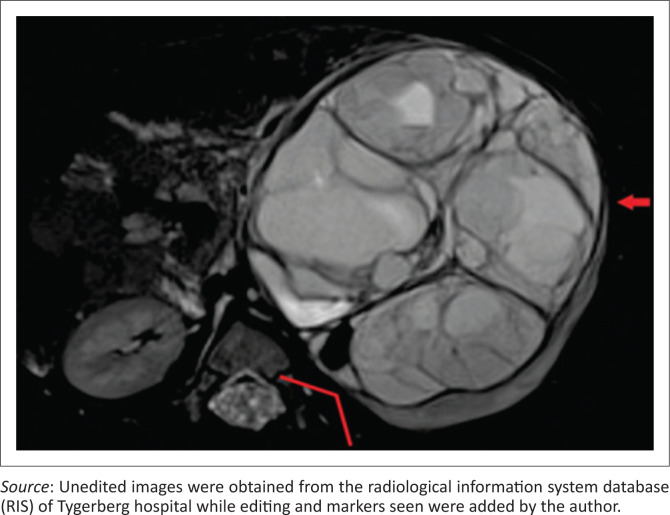
Axial fat-suppressed T2-weighted magnetic resonance image of a large cystic nephroblastoma arising from the left kidney. The claw sign is positive with tapering (red arrow), absent infolding and obtuse external kidney–tumour interface (red marker).

**FIGURE 4:**
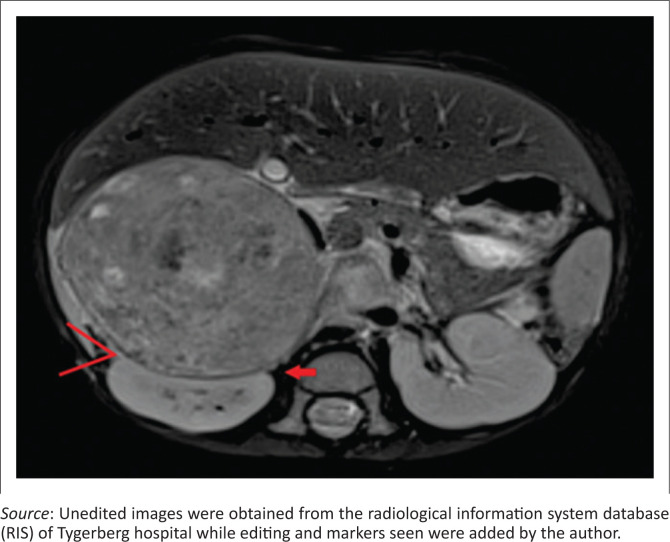
Axial fat-suppressed T2-weighted magnetic resonance image of a neuroblastoma compressing the right kidney. The claw sign is negative with absent tapering, infolding (red arrow) and acute external kidney–tumour interface.

**FIGURE 5:**
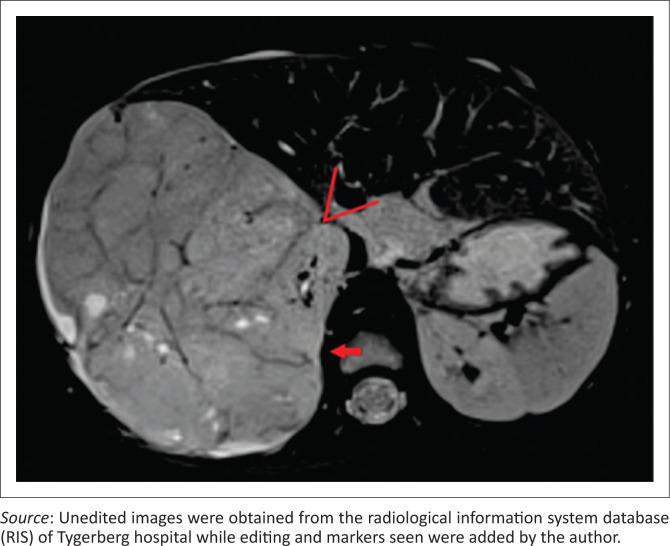
Axial fat-suppressed T2-weighted magnetic resonance image of a large nephroblastoma arising from the inferior pole of right kidney. The claw sign is positive at the posterior kidney–tumour interface (red arrow). However, there is infolding and an acute angle at the anterior kidney–tumour interface (red marker). In conflicting cases, a positive claw sign was diagnosed.

Clear positive and negative claw signs are illustrated in Figures 3 and 4, respectively. The imaging findings may, however, be conflicting particularly with a large tumour size, wherein all three signs may not be simultaneously present or may in fact be in conflict (Figure 5). In this study, where conflicting imaging findings were encountered between axial and coronal images, a consensus decision was made to award a positive claw sign in the presence of a tapered kidney edge in either plane. This sign demonstrated the best sensitivity (97%), specificity (74%), positive predictive value (83%) and negative predictive value (94%) overall.

The following unambiguous definition of the claw sign is therefore proposed: A claw sign can be regarded as positive when a renal mass displays a tapered kidney edge, supported by absent infolding of the kidney edge and an obtuse external kidney–tumour interface.

This study had limitations regarding the study population. All efforts were made to obtain complete results in order to maximise the study population. However, if the study criteria were not met, cases were excluded from the study.Undoubtedly a larger study population would offer more accurate and complete results as further discussed in relation to the Cohen’s kappa analysis. The most pressing statistical concern is also the most fundamental under the frequentist approach. The small sample size (53 cases) raises questions about the reliability of many of the assumptions upon which the Cohen’s kappa coefficients and their respective CIs are built.

A concern relating specifically to the Cohen’s kappa analysis is the comparative approach to inter-rater reliability with respect to the supposed ‘random’ chance of agreement between the two interpreters (radiologists).^9^ One does have to consider the possibility that comparison to a completely random possibility of agreement between two qualified professionals, who are performing a diagnosis within their field of expertise, is not completely accurate. One expects some degree of agreement between two doctors applying a standard set of skills to the same problem. Further general pitfalls include the limited number of inter-rater parties that one is able to evaluate using Cohen’s kappa coefficient.^9^

## Conclusion

The claw sign can be defined as a mass with a characteristic tapered kidney edge that may be supported by absent infolding of the kidney edge and an obtuse external kidney–tumour interface. No clear preference for axial or coronal MRI plane for assessment of the sign has been demonstrated. Intra- and inter-rater reliability is moderate to strong for all the above-mentioned characteristics and the overall impression of the claw sign. The claw sign is highly sensitive but lacks specificity.

The tumour size at which the claw sign most optimally performs could not be established. Larger studies are needed to assess this.
